# Worries of Pregnant Women: Testing the Farsi Cambridge Worry Scale

**DOI:** 10.1155/2016/5791560

**Published:** 2016-05-17

**Authors:** Forough Mortazavi, Arash Akaberi

**Affiliations:** ^1^Reproductive Health, Education Development Center, Sabzevar University of Medical Sciences, Sabzevar 9613873136, Iran; ^2^Department of Epidemiology and Biostatistics, Sabzevar University of Medical Sciences, Sabzevar 9613873136, Iran; ^3^School of Continuing Studies, McGill University, Montreal, QC, Canada H3H 2A2

## Abstract

Pregnancy adds many sources of concerns to women's daily life worries. Excessive worry can affect maternal physiological and psychological state that influences the pregnancy outcomes. The aim of this study was to validate the Cambridge Worry Scale (CWS) in a sample of Iranian pregnant women. After translation of the CWS, ten experts evaluated the items and added six items to the 17-item scale. In a descriptive cross-sectional study, 405 of pregnant women booked for prenatal care completed the Farsi CWS. We split the sample randomly. Exploratory factor analysis (EFA) was conducted on the first half of the sample to disclose the factorial structure of the 23-item scale. The results of the EFA on the Farsi CWS indicated four factors altogether explained 51.5% of variances. Confirmatory factor analysis (CFA) was done on the second half of the sample. The results of the CFA showed that the model fit our data (chi-square/df = 2.02, RMSEA = 0.071, SRMR = 0.071, CFI = 0.95, and NNFI = 0.94). Cronbach's alpha coefficient for the Farsi CWS was 0.883. The Farsi CWS is a reliable and valid instrument for understanding common pregnancy worries in the third trimester of pregnancy in Iranian women.

## 1. Introduction 

Pregnancy is a period in a woman's life filled with pleasant anticipation of a baby, which may be influenced by several psychological stressors. Research on the psychological state in pregnancy indicates that each trimester of pregnancy includes possible stressors that may provoke several worries for pregnant women [1]. Some studies found heightened levels of worries in the first and third trimester of pregnancy [2] whereas others showed that each facet of a pregnant woman's worries may fluctuate across the course of pregnancy [3, 4]. Several studies indicated that worries about the possibility of losing the baby, baby's health, and childbirth are common causes of concerns among pregnant women [1, 3, 5–7]. In addition to this kind of worries, there are other sources of worries in women daily life (e.g., worry about money, job, housing, their health, and marital relationships) [8]. Most women experience some mild worries during pregnancy; however, some women may experience pathological worries, defined as uncontrollable negative thoughts and excessive concern about future events in pregnancy which can produce anxiety [9].

Women with pregnancy-related anxiety may experience symptoms such as muscle pain, palpitation, fatigue, headaches, stomach pain, sleep disruption, nightmares, and insomnia [10, 11] which can influence maternal health and quality of life. Maternal anxiety has been a risk factor for poor perinatal outcomes such as preterm birth [12–14], postpartum depression [15, 16], caesarean [13, 17], and poor child developmental outcomes [13]. Thus, it is important to measure the extent and content of anxiety and worry during the course of pregnancy.

The State-Trait Anxiety Inventory (STAI) has been a widely used instrument in the area of pregnancy-related anxiety and worry [16, 18]. There are problems using the STAI as a measure of pregnancy-related anxiety and worry. The first is that the STAI can only measure the severity of anxiety and cannot reveal the reasons for anxiety [4]. The second problem is that the STAI measures general anxiety while it is probable that a mother who is neither typically depressed nor presently anxious for other reasons be worried about her baby's health or childbirth. Result of a study found that general anxiety and depression explained 8–27% of the variation in worries related to the fetal health and childbirth in the first and second trimester of pregnancy [19].

Moreover, although worry and anxiety are closely related to each other [20], there are differences between the two concepts [21, 22]. Anxiety is composed of cognitive, somatic, emotional, and behavioral elements [20], whereas worry is specified as the cognitive component of anxiety with a significant positive effect on it [22]. Cognitive dimensions of pregnancy-related anxiety include fetal health, loss of fetus, childbirth, mother's well-being, body image, parenting and care for child, health care related, financial, and family and social support [11]. Thus, a specific instrument is needed to measure worry in pregnant women.

Since the recognition of excessive uncontrollable worry as a main feature of generalized anxiety disorder in the DSM-IV [23], measures of worry have been developed focusing on the intensity of worry [24] and areas of worry [25]. For those grounds, Statham et al. developed the Cambridge Worry Scale (CWS) to investigate the prevalence and content of worries during pregnancy. The 17-item CWS included items that assessed both pregnancy-related worries and daily life worries. They examined the CWS in “the Cambridge Prenatal Screening Study,” a longitudinal study on 1072 pregnant women in which women's worries were assessed in 16-, 22-, and 35-week gestation [3]. In a validation study, they examined the CWS on 1207 pregnant women. A four-factor structure of pregnant women's worries was found (sociomedical, own health, socioeconomic, and relational). The scale demonstrated good reliability and validity and the CWS subscales were correlated with state and trait anxiety [4].

The CWS has been translated into Turkish, Spanish, German, Swedish, and Greek and the reliability and the content validity of the translated scales were confirmed [1, 6, 7, 26, 27]. Despite the use of the CWS in different populations, the test has not been used in Iranian women. The aim of the present study was to translate and investigate the reliability and validity of the CWS in Iran. We also aimed to explore the prevalence of common worries among pregnant women in Sabzevar. To our knowledge, no study has validated the CWS in pregnant women in Iran.

## 2. Material and Methods

This was a descriptive cross-sectional study. The sample included 405 pregnant women who were registered for receiving prenatal care in eight health clinics affiliated with Sabzevar University of Medical Sciences, Iran, in 2014. To select clinics, the city was divided into four regions. In each area, two clinics were randomly selected. In each clinic, all pregnant women who consented to participate in the study and met the criteria were enrolled. The inclusion criteria were as follows: having the ability to read and write and being in the third trimester of pregnancy. Women who suffered from psychological problems were excluded from the study. The women completed a questionnaire consisting of sociodemographic and obstetrical information, the Farsi CWS, Childbirth Attitudes Questionnaire (CAQ), and the State-Trait Spielberger scale (STAI).

### 2.1. Instruments

#### 2.1.1. Childbirth Attitudes Questionnaire (CAQ)

Harman created the Childbirth Attitudes Questionnaire (CAQ) [28]. Lowe revised it, added a summary question to the scale, and supported reliability and validity of the scale [29]. The scale included 16 items with a response scale of 1–4 with higher scores indicating higher childbirth fear. The scale is unidimensional. Items begin with “fear of” such as “fear of the baby being injured during childbirth,” “fear of being torn during childbirth,” “fear of having to have a caesarean section,” and “fear of something being wrong with the baby.” The internal consistency reliability of the scale measured by Cronbach's alpha was 0.83 [29]. The Farsi CAQ consisted of 14 items with total points of 14–56. Khorsandi et al. supported the content validity of the CAQ. The internal consistency of the questionnaire was good (Cronbach's alpha = 0.84) [30].

#### 2.1.2. The State-Trait Anxiety Inventory (STAI)

The Spielberger State-Trait Anxiety Inventory (STAI) [31] includes two inventories which measure state anxiety that can fluctuate over time and trait anxiety that is anxiety level as a personal characteristic which is stable over time. Each scale is composed of 20 items. Items are scored on a 4-point Likert scale ranging from 1 to 4. The total score ranges from 20 to 80 for each scale, with a higher score indicating higher anxiety [31]. The measures demonstrated excellent internal consistency (Cronbach's alpha = 0.89) [32]. Mahram translated the scale into Farsi and supported the validity of the instrument. The internal consistency of the state and trait subscales measured by Cronbach's alpha was 0.91 and 0.90, respectively [33].

#### 2.1.3. Cambridge Worry Scale (CWS)

Cambridge Worry Scale (CWS) developed by Green et al. is a questionnaire that consists of 17 items. The 17-item CWS measures the severity of common pregnant worries. Scores are ranged from “not a worry” (0) to “major worry” (5). The total score can be 0 to 85, with a higher score representing the severity of worries. The scale includes four subscales as follows: sociomedical, own health, socioeconomic, and relational. Content validity and internal consistency of this scale at 34 weeks were demonstrated during early development of the CWS (Cronbach's alpha = 0.76) [4]. The CWS has been translated and validated into Turkish, Spanish, German, Swedish, and Greek [1, 6, 7, 26, 27] and its reliability and validity were supported. The construct validity of the Spanish, German, and Greek versions of the scale was confirmed [6, 7, 27].

### 2.2. Process of Translation

The CWS was translated into Farsi and back-translated into English by two specialists in the English language. A bilingual Ph.D. compared the three versions. Few minor revisions were done.

### 2.3. Content Validity

An expert panel that consisted of four faculty members and specialists of reproductive health, gynecology, and midwifery and six midwives who had worked in the antenatal clinics at least for 15 years evaluated the items and discussed their relevance to the Iranian culture. Since worries towards pregnancy depend largely on culture, facilities, and the environment in which women become pregnant and give birth, experts discussed adding six items to the scale based on their experiences. Those items include baby gender, unplanned or unwanted pregnancy, the probability of not having a spontaneous labor, crowded delivery ward, whether midwives provide good care in labor, and not having someone in delivery ward. In addition, they changed the item “whether your partner will be with you for the birth” to “whether your husband will be with you at the time of admission to labor” since men are not permitted to be in labor rooms in most hospitals in Iran.

To determine content validity ratio (CVR), we chose Lawshe method [34]. In the quantitative phase of the content validity, experts assessed the necessity of the items using a three-point rating scale: (a) not necessary, (b) useful, but not essential, and (c) essential. The CVR for every item was calculated. No item had a CVR less than 0.62 (acceptable CVR value for ten experts).

In the next step, experts judged the clarity, simplicity, and relevance of each item on a 4-point Likert scale (a = not relevant, not simple, and not clear to d = very relevant, very simple, and very clear). The content validity index (CVI) for every item was calculated by dividing the total number of experts by the number of experts who had chosen the (c) or (d) option for each particular item. We calculated the CVI for relevance, clarity, and simplicity of every item. No item had a CVI less than 0.8 which is recommended by Polit and Beck as the acceptable lower limit for the CVI value [35].

In the pilot study, we asked 20 pregnant women to fill out the Farsi CWS to assess if they felt the items were relevant to a pregnant mother's worries and if they felt difficulty in responding to the items. Most women indicated that the questionnaire was understandable. We also asked them to rate the importance of each item on a 5-point Likert scale (1 = not important to 5 = very important). We calculated an impact score for each item [36]. The impact score of all items was ≥1.5.

### 2.4. Statistical Analysis

Data analyses were performed by SPSS v. 18 (SPSS, Inc., Chicago, IL, USA). Cronbach's alpha coefficient was used to investigate the reliability of the Farsi CWS. Cronbach's alpha values > 0.7 were considered acceptable [37]. Since new items were added to the scale, it was necessary to investigate the factorial model of the 23-item scale. The sample was randomly split into two halves. Exploratory factor analysis (EFA) was conducted on the first half of the sample to disclose the factorial structure of the 23-item scale. The extraction method was principal component analysis and the rotation method was oblimin with Kaiser normalization. Factor loading > 0.3 was considered acceptable loading. The number of factors was obtained by the scree plot. Confirmatory factor analysis (CFA) was run on the second half of the sample and based on the model extracted by the EFA. We considered a relative chi-square < 3.00 [38], a root mean square error of approximation (RMSEA) value of <0.08, a comparative fit index (CFI) value of 0.90≤ [39], and a standardized root mean square residual (SRMR) value of <0.08 [40] as acceptable model fit.

Concurrent validity was examined by calculating Spearman correlation coefficients between the CWS and STAI. For discriminant validity, the Farsi CWS scores of women with low and high childbirth fear were compared using a *t*-test. The median CAQ score of 37 was used as a cutoff point for childbirth fear in this study. Predictive validity was examined using a *t*-test by comparing the mean score of the Farsi CWS in women who preferred a caesarean with that of women who preferred a vaginal delivery.

## 3. Results 

### 3.1. Subjects

**Figure 1:**
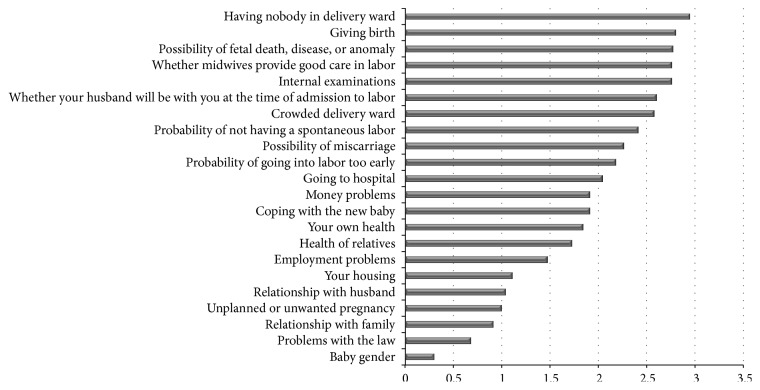
Ranking of women's worries.

Women's characteristics are presented in Table 1. The mean of women's age was 26.3 ± 5.1 years. Weeks of gestation ranged from 28 to 40 weeks. The mean gestational age was 34.6 ± 3.2 weeks. Eighty-five percent of women were homemakers and only 4% were self-employed. Forty-two percent of women were multiparous. In Table 2, descriptive parameters of the Farsi CWS items are displayed. The first seven worries belong to the sociomedical subscale and all are related to childbirth. In Figure 1, the mean score of each item is presented.

### 3.2. Validity

#### 3.2.1. Exploratory Factor Analysis (EFA)

Since new items were added to the scale, an EFA was used to reveal the factorial structure of the Farsi 23-item CWS. The results of the EFA on the 23-item Farsi CWS and on the first half of the sample revealed four factors, altogether explaining 51.5% of variance (Table 3). The first factor explained 29.5% of variance. One item (giving up work) was not loaded on any factor that was removed from the scale. Correlations between the Farsi CWS and its subscales are 0.39–0.86, indicating moderate correlation and acceptable convergent validity (Table 4).

#### 3.2.2. Confirmatory Factor Analysis (CFA)

**Figure 2:**
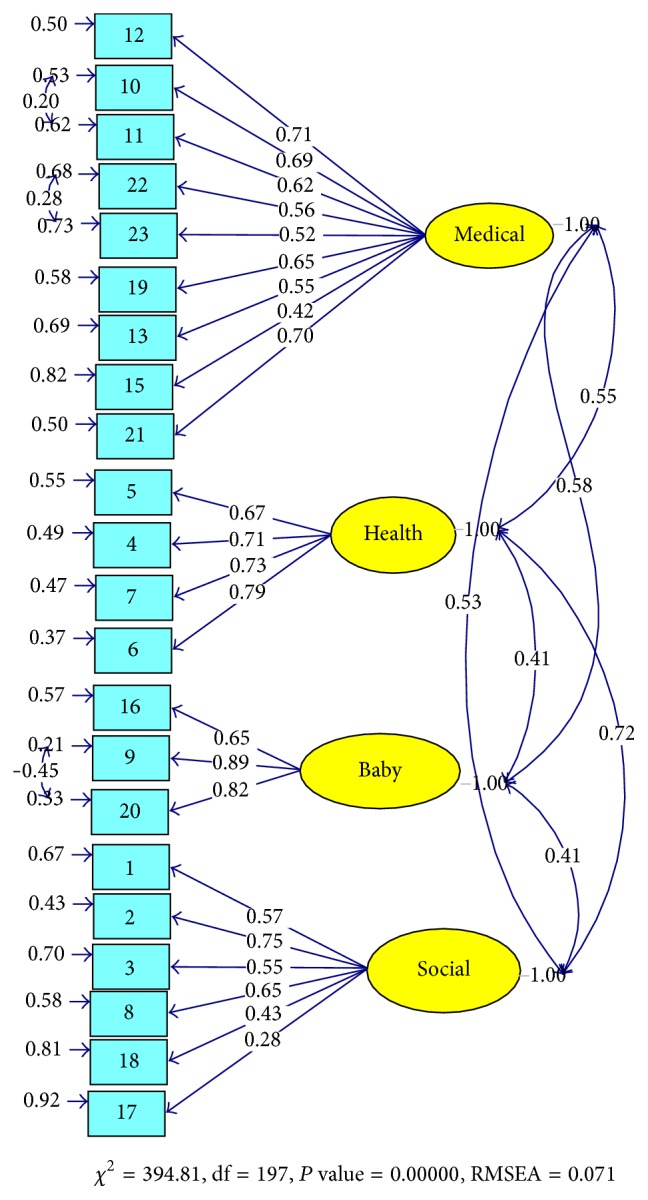
The results of CFA on the 22-item Cambridge Worry Scale.

Results of the CFA on 22 items and on the second half of the sample revealed that the four-factor model yielded by the EFA was acceptable (chi-square/df = 2.02, RMSEA = 0.071, SRMR = 0.071, CFI = 0.95, IFI = 0.95, and NNFI = 0.94).  Figure 2 displays the results of the CFA.

#### 3.2.3. Concurrent Validity

The correlation coefficients between the 22-item Farsi CWS scores and STAI-State, STAI-Trait, and CAQ scores were 0.54, 0.49, and 0.54, respectively, confirming concurrent validity (*P* < 0.001) (Table 4).

#### 3.2.4. Discriminant Validity

Results showed that women with higher childbirth fear (CAQ scores > 37) had higher total worry scores than women with lower childbirth fear (CAQ scores < 37) (Table 5). All items except “housing problems” could differentiate between women with high childbirth fear and those with low fear (*P* < 0.001). The mean scores of the items of the health of a mother and socioeconomic subscales (*P* < 0.001) could differentiate between women with unwanted and wanted pregnancy. The mean scores of items “money problems,” “the possibility of fetal disease,” “coping with the new baby,” “whether your husband will be with you at the time of admission to labor,” “possibility of miscarriage,” and “not possible to have someone in delivery ward” were higher in younger women (<30) than elders (*P* < 0.05). The mean scores of items “money problems,” “problems with the law,” and “relationship with husband” were higher in low educated women (<12 years) than women with an academic educational background (*P* < 0.05).

#### 3.2.5. Predictive Validity

We also evaluated the construct validity by determining the predictive validity of the 22-item Farsi CWS. In addition to the sociomedical subscale mean score (Table 5), all the items of this subscale could differentiate between women who preferred caesarean and those who preferred normal delivery (*P* < 0.001).

### 3.3. Reliability

Table 6 shows Cronbach's alpha coefficients for the four subscales of the 22-item Farsi CWS. There was no difference in Cronbach's alpha coefficients for the CWS in primiparas and multiparas.

## 4. Discussion 

This study was the first to describe the validity and reliability of the Farsi CWS in pregnant women in Iran. The CWS assesses all kinds of worries, which a pregnant woman may experience, either related to pregnancy or related to her daily life. The results indicate that the 22-item Farsi CWS version is a reliable and valid instrument for measuring worries in pregnant Iranian women in the third trimester of pregnancy.

Internal consistency of the 22-item Farsi CWS (0.88) and its subscales was satisfactory which chimes with previous studies [4, 6, 7, 26, 27]. The EFA on the 22-item scale indicated a four-factor structure for the Farsi CWS consisted of (1) sociomedical, (2) health of mother/others and relationships, (3) baby's health, and (4) socioeconomic factors. Results showed that the first factor explained about 30% of the variance. The explained variance indicates that the loadings of the items in the first factor are high, which means it is an important factor in describing the relationship between the variables. The structure we found in this study did not match up completely with the structure found in Green's study. The structure that Green found consisted of four factors including (1) sociomedical, (2) socioeconomic, (3) health, and (4) relationship factors. The structures found in two other studies were not the same as the original structure either [7, 26]. In Petersen et al.'s study, four factors were found as follows: (1) sociomedical, (2) socioeconomic and relationships, (3) health of the baby, and (4) health of mother/others. Like our study, they found that the sociomedical factor explained a large percentage of the variance (about 27%). They also found two distinct health factors: the health of the baby and the health of mother/others [7]. In Gunay and Gul's study, the Turkish form of the CWS was found to be appropriate in terms of language and content validity, but no factorial structure for the scale was confirmed [26]. Jomeen and Martin's study discovered a structure similar to the structure which was found in Green's study with two items related to more than one latent variable [41]. In three studies, a four-factor structure similar to the structure found in Green's study emerged with different factor orders [6, 27, 42].

Regarding the discriminant validity, the Farsi 22-item CWS performed well. It was demonstrated that women with fear of childbirth would experience more worries. Also, almost all items of the scale could differentiate between women with high and low childbirth fear. Moreover, six items could discriminate between younger and older women.

Concurrent validity was also confirmed by the moderate correlations between the scores of the CWS and STAI which meant that the Farsi CWS measured something different from the STAI and scores are not just a reflection of tendency to worry about all things. In previous studies, similar moderate correlations were found between the CWS and STAI [4, 6, 7].

With relation to the predictive validity of the Farsi CWS, we found higher mean scores of the sociomedical items in women who preferred caesarean than women who preferred a vaginal delivery, which is in agreement with the results of previous studies [13, 29].

In this study, the most prevalent causes of worries in pregnant women were having nobody in delivery ward, giving birth, and the possibility of something being wrong with the baby. Although, in private hospitals or big university hospitals in major cities in Iran, it is possible to have a woman in the labor room, there is one maternity hospital in Sabzevar and women are alone during the labor and delivery. In our study, baby's health was the third important worry. In previous studies, worry that something might be wrong with the baby was the most important worry [3, 4, 6].

We translated the CWS into Farsi and validated it. Our scale consisted of 22 items while in previous studies the number of items reduced to 13 items [4, 7, 27]. This is due to the differences between the settings and environment in which Iranian and European mothers give birth. In addition, item 14 (giving up work) was not loaded on any factor. Since most employed women were on maternity leave at the time of sampling, the item “giving up work” was not suitable for them. This item was excluded in previous studies [4, 7, 27].

The revised item “whether your husband will be with you at the time of admission to labor” remained in the Farsi CWS scale, indicating that this is an important worry in Iranian pregnant women. Previous qualitative study in Iran showed that women were interested in having their husband during childbirth [43]. In Green et al.'s study, item “whether your partner will be with you for the birth” was removed from the scale due to low communality in the EFA [4].

In our study, baby gender and unplanned/unwanted pregnancy were expressed by a low percentage of women as a source of worry. That might be due to the fact that the crises of fetal gender or unplanned/unwanted pregnancy are usually resolved till the third trimester of pregnancy [44]. Therefore, assessing the psychometric properties of the CWS on women in the first and second trimester of pregnancy would probably lead to somewhat different findings. We recommend that in future validation studies the scale in the first and second trimesters of pregnancy be examined.

### 4.1. Implications for Practice and Policy

The Farsi 22-item version CWS is a short, simple, and appropriate instrument for understanding women's worries in the third trimester of pregnancy in Iran.

### 4.2. Limitations and Strengths

The strength point of the study is its sample size that enabled us to split it for assessing the construct validity. The weak point of the study was that we limited our study to a specific trimester of pregnancy. Since the extent and content of worries may differ in the three trimesters of pregnancy, it may influence the results of the study leading to low level of worry expressed by women about baby gender and unwanted/unplanned pregnancy or highlighted women's worry about giving birth.

### 4.3. Conclusion

The present study confirmed the content validity, reliability, and construct validity of the Farsi CWS. Results of this study indicated that the sociomedical items including not having someone in delivery ward, giving birth, and the possibility of something being wrong with the baby were the most prevalent causes of worry in pregnant women.

## Figures and Tables

**Table 1:** Women's characteristics.

Variables	*N* (%)
Age (years)	
<20	56 (14)
20–30	235 (59)
30<	107 (27)
Educational level (years)	
<6	16 (4)
6–12	233 (58)
12≤	151 (38)
Job	
Homemaker	340 (85)
Employed	31 (7.5)
Self-employed	16 (4.0)
Student	14 (3.5)
Parity	
None	234 (58)
One	136 (34)
Two or more	34 (8)
History of abortion	
Yes	68 (17)
No	337 (83)
Income (million RIL)	
<10	296 (75)
10–20	87 (22.2)
20≤	14 (2.8)

**Table 2:** Descriptive parameters of the Farsi Cambridge Worry Scale.

	Not a worry %	1 %	2 %	3 %	4 %	Major worry %	% of 4 and 5	Mean
Having nobody in delivery ward	15.4	15.6	8.3	9.8	20.9	30.0	50.9	2.95
Giving birth	12.1	19.4	9.6	17.4	18.9	22.7	41.6	2.80
Possibility of fetal death, disease, or anomaly	16.6	20.7	8.1	8.8	15.9	30.0	45.9	2.77
Whether midwives provide good care in labor	9.6	20.7	12.8	16.9	20.9	19.1	40.0	2.76
Internal examinations	16.9	18.6	10.1	11.6	19.6	23.2	42.8	2.68
Whether your husband will be with you at the time of admission to labor	19.6	20.2	8.1	9.8	17.6	24.7	42.3	2.60
Crowded delivery ward	16.6	20.9	7.1	15.6	22.4	17.4	39.8	2.58
Probability of not having a spontaneous labor	16.6	23.7	11.1	14.9	18.6	15.1	33.7	2.41
Possibility of miscarriage	34.3	15.6	4.8	8.1	10.1	27.2	37.3	2.26
Probability of going into labor too early	27.0	19.7	9.1	11.4	18.4	14.4	32.8	2.18
Going to hospital	25.7	23.2	9.8	14.9	15.9	10.6	36.5	2.04
Money problems	29.5	22.2	9.6	14.9	14.4	9.6	24.0	1.91
Coping with the new baby	25.4	26.4	10.8	14.9	13.9	8.6	22.5	1.91
Your own health	24.9	32.2	9.6	10.1	13.1	10.1	23.2	1.84
Health of relatives	31	25.4	13.6	8.8	11.6	9.6	21.2	1.73
Employment problems	45.8	16.9	8.3	10.6	10.3	8.1	18.4	1.47
Relationship with husband	56.9	17.6	7.1	6.8	6.5	5.0	11.5	1.04
Your housing	47.6	26.7	6	9.8	6	3.8	9.8	1.11
Unplanned or unwanted pregnancy	84.6	7.8	1.5	2.5	1.5	2	3.5	1.0
Relationship with family	57.7	18.6	9.8	6.5	3.8	3.5	7.3	0.91
Problems with the law	68.5	13.9	6.5	5.3	3.8	2.0	5.8	0.68
Baby gender	63.5	13.1	3.8	7.3	4.8	7.6	12.4	0.35

**Table 3:** The Farsi Cambridge Worry Scale four factors and factor loadings (22-item version).

Items	Factors loading				Communality
	Sociomedical	Health of mother & relationships	Health of the baby	Socioeconomic	
*Sociomedical *					
(12) Giving birth	0.832				0.692
(10) Going to hospital	0.711				0.594
(11) Internal examinations	0.699				0.534
(22) Crowded delivery ward	0.679				0.569
(23) Not possible to have someone in delivery ward	0.642				0.575
(19) Whether midwives provide good care in labor	0.671				0.485
(13) Coping with the new baby	0.497				0.392
(21) The possibility of not having a spontaneous labor	0.484		*0.376*		0.491
(15) Whether your husband will be with you at the time of admission to labor	0.463				0.397
*Health of mother/others & relationships *					
(5) Your relationship with your family and friends		0.747			0.661
(4) Your relationship with your husband		0.742			0.733
(7) The health of someone close to you		0.484			0.574
(6) Your own health		0.430	*0.301*	*0.304*	0.495
*Health of the baby*					
(16) Possibility of miscarriage			0.805		0.640
(9) The possibility of fetal death, disease, or anomaly			0.786		0.669
(20) Probability of going into labor too early			0.657		0.503
*Socioeconomic *					
(1) Your housing				0.607	0.352
(2) Money problems				0.625	0.556
(3) Problems with the law				0.608	0.484
(8) Employment problems				0.540	0.404
(18) Unwanted or unplanned pregnancy				0.525	0.243
(17) Baby gender				0.399	0.189
Eigenvalue	6.5	2.2	1.5	1.2	—
Variance %	29.5	9.8	6.7	5.5	—

Kaiser-Meyer-Olkin test = 0.831 and Bartlett's test = 1559 (*P* < 0.001); total variance explained: 51.5%.

**Table 4:** Correlation matrix of the subscales of the 22-item Farsi CWS^†^, CAQ^‡^, and STAI^§^.

	1	2	3	4	5	6	7	8
(1) CWS	1							
(2) Sociomedical	0.86^*∗∗*^	1						
(3) Health of mother/others & relationships	0.73^*∗∗*^	0.47^*∗∗*^	1					
(4) Health of baby	0.69^*∗∗*^	0.46^*∗∗*^	0.42^*∗∗*^	1				
(5) Socioeconomic	0.72^*∗∗*^	0.43^*∗∗*^	0.57^*∗∗*^	0.39^*∗∗*^	1			
(6) CAQ	0.53^*∗∗*^	0.64^*∗∗*^	0.27^*∗∗*^	0.30^*∗∗*^	0.22^*∗∗*^	1		
(7) State anxiety	0.54^*∗∗*^	0.47^*∗∗*^	0.39^*∗∗*^	0.22^*∗∗*^	0.42^*∗∗*^	0.47^*∗∗*^	1	
(8) Trait anxiety	0.49^*∗∗*^	0.53^*∗∗*^	0.39^*∗∗*^	0.24^*∗∗*^	0.39^*∗∗*^	0.50^*∗∗*^	0.86^*∗∗*^	1
(9) Age	−0.085	−0.067	−0.063	−0.036	−0.095	0.004	0.02	0.05

^*∗∗*^ *P* < 0.01, ^†^Cambridge Worry Scale, ^‡^Childbirth Attitudes Questionnaire, and ^§^State-Trait Anxiety Inventory.

**Table 5:** Means of the 22-item Farsi Cambridge Worry Scale scores based on sociodemographic variables.

Variable	Scale factors						
		*N*	Total worry score	Sociomedical	Health of mother/others & relationships	Baby's health	Socioeconomic
Age	<30	291	40.2 ± 18.4	20.7 ± 9.6	5.59 ± 4.9	7.5 ± 4.5	5.5 ± 4.5
	30≤	107	35.4 ± 21.4	18.2 ± 10.0	5.2 ± 5.2	6.3 ± 5.0	4.2 ± 4.2
*P*			0.021^*∗*^	0.027^*∗*^	0.540	0.039^*∗*^	0.054
Education	≤12	243	40.2 ± 19.6	20.0 ± 9.8	5.9 ± 5.2	7.4 ± 4.7	7.0 ± 5.6
	12<	147	37.3 ± 18.9	20.2 ± 9.7	4.8 ± 4.5	6.7 ± 4.7	5.6 ± 5.4
*P*			0.151	0.813	0.032^*∗*^	0.184	0.013^*∗∗*^
Unwanted/unplanned pregnancy	No	278	37.2 ± 18.5	19.7 ± 9.9	4.9 ± 4.5	7.1 ± 4.7	5.6 ± 5.0
	Yes	117	44.0 ± 20.3	21.0 ± 9.3	7.0 ± 5.6	7.4 ± 4.7	8.7 ± 6.3
*P*			0.001^*∗∗*^	0.243	<0.001^*∗∗∗*^	0.471	<0.001^*∗∗∗*^
CAQ score^†^	<37^‡^	179	30.2 ± 15.9	14.5 ± 7.9	4.2 ± 4.3	6.2 ± 4.4	5.3 ± 5.6
	37≤	218	47.1 ± 18.5	24.9 ± 8.5	6.6 ± 5.2	8.1 ± 4.8	7.5 ± 5.3
*P*			<0.001^*∗∗∗*^	<0.001^*∗∗∗*^	<0.001^*∗∗∗*^	<0.001^*∗∗∗*^	<0.001^*∗∗∗*^
Request for caesarean	Yes	65	44.2 ± 19.6	24.3 ± 9.5	5.8 ± 4.9	7.4 ± 5.0	6.8 ± 5.6
	No	335	38.3 ± 19.0	19.3 ± 9.6	5.4 ± 5.0	7.1 ± 4.6	6.5 ± 5.6
*P*			0.022^*∗*^	<0.001^*∗∗∗*^	0.602	0.702	0.693

^*∗*^ *P* < 0.05, ^*∗∗*^ *P* < 0.01, ^*∗∗∗*^ *P* < 0.001, ^†^Childbirth Attitudes Questionnaire score, and ^‡^<37 indicative of low childbirth fear.

**Table 6:** Cronbach's alpha coefficients of the Farsi Cambridge Worry Scale.

Subscales	22-item version		
	Total	Primiparas	Multiparas
Sociomedical (9 items)	0.847	0.845	0.847
Health of mother/others & relationships (4 items)	0.803	0.798	0.810
Health of the baby (3 items)	0.715	0.689	0.744
Socioeconomic (6 items)	0.690	0.684	0.695
Total	0.886	0.883	0.891
